# Research Progress of Electrically Driven Multi-Stable Cholesteric Liquid Crystals

**DOI:** 10.3390/ma17010136

**Published:** 2023-12-27

**Authors:** Kainan Wang, Wentuo Hu, Wanli He, Zhou Yang, Hui Cao, Dong Wang, Yuzhan Li

**Affiliations:** School of Materials Science and Engineering, University of Science and Technology Beijing, Beijing 100083, China

**Keywords:** electrical driving mode, multi-stability, liquid crystals, cholesteric phase, optical devices, bistable mode, tri-stable mode, multi-stable mode

## Abstract

Electrically driven multi-stable cholesteric liquid crystals can be used to adjust the transmittance of incident light. Compared with the traditional liquid crystal optical devices, the multi-stable devices only apply an electric field during switching and do not require a continuous electric field to maintain the various optical states of the device. Therefore, the multi-stable devices have low energy consumption and have become a research focus for researchers. However, the multi-stable devices still have shortcomings before practical application, such as contrast, switching time, and mechanical strength. In this article, the latest research progress on electrically driven multi-stable cholesteric liquid crystals is reviewed, including electrically driven multi-stable modes, performance optimization, and applications. Finally, the challenges and opportunities of electrically driven multi-stable cholesteric liquid crystals are discussed in anticipation of contributing to the development of multi-stable liquid crystal devices.

## 1. Introduction

Cholesteric liquid crystals (ChLCs) are the earliest discovered and commonly used mesophase, usually presenting colorful appearances [1,2]. When observed under a polarizing microscope, cholesteric liquid crystals often exhibited planar texture, focal conic texture, fingerprint texture, and homeotropic texture. Planar texture is usually a transparent state with selective reflection of the incident light. In the focal conic texture, due to the random distribution of the ChLC spiral axis, the incident light is scattered and the device appears opaque. When in the fingerprint texture, the spiral axis of the liquid crystal is arranged parallel to the substrate and cannot selectively reflect the wavelength of the incident light, so the device is in a transparent state. In the homeotropic texture, the direction order of liquid crystal molecules is arranged perpendicular to the substrate and can be used for transitions to other textures, and the ChLC devices in this texture are usually transparent. ChLCs have a sensitive electric field response, diverse textures, and corresponding optical states, which can be used in different optical applications especially, and electrically driven optical devices are one of the key research directions [3,4,5,6,7].

As is well known, the directional vector of liquid crystal molecules with dielectric anisotropy can be changed within a few milliseconds under an external electric field. When the directors are chaotic, the incident light is scattered. On the contrary, when the directors are uniform, the incident light can be transmitted. Electrically driven cholesteric liquid crystal devices can be broadly divided into mono-stable cholesteric liquid crystal devices and multi-stable cholesteric liquid crystal devices. The stable state refers to the state of the device stabilized in the absence of external stimuli. Compared with mono-stable devices [8,9,10,11,12,13,14], multi-stable devices can be stabilized in a variety of states (mostly two or three) when there is no external stimulus [15,16,17,18,19,20]. Due to the low energy consumption (especially when the device does not need to frequently switch its optical state), multi-stable devices have strong application prospects in energy-saving places [21,22,23]. Therefore, multi-stable direction is a key research direction in the field of optical devices. At present, electrically driven multi-stable cholesteric liquid crystal devices have mainly developed into bistable mode, tri-stable mode, and multi-stable mode. In bistable mode, the most common switching mode is between the planar texture and the focal conic texture. In addition, switching between homeotropic texture and focal conic texture can be realized by introducing a liquid crystalline polymer network. In tri-stable mode, ChLCs can be switched between planar texture, focal conic texture, and fingerprint texture under an external electric field, while the multi-stable mode is more complex and challenging to develop, such as designing an additional state with wider wavelength reflection based on the tri-stable mode. At present, some progress has been made in electrically driven multi-stable cholesteric liquid crystal optical devices. Therefore, it is necessary to summarize and review them in order to facilitate subsequent research as well as promote their development.

## 2. Electrically Driven Multi-Stable Modes of Cholesteric Liquid Crystals

### 2.1. Bistable Mode

In bistable mode, devices have two states: transparent and opaque. The liquid crystal molecules under the two states tend to have lower free energy so that the two states can be stabilized for a long time. Depending on the different dielectric anisotropy (∆ε), the liquid crystal molecules in the bistable mode have three cases under an applied electric field. Liquid crystal (Δε>0) molecules with positive dielectric anisotropy are aligned parallel to the direction of the electric field and liquid crystal (Δε<0) molecules with negative dielectric anisotropy are aligned perpendicular to the direction of the electric field. In addition to positive and negative dielectric liquid crystals, dual-frequency liquid crystals can switch between positive dielectric anisotropy and negative dielectric anisotropy under the low- and high-frequency electric fields [24].

**Figure 1:**
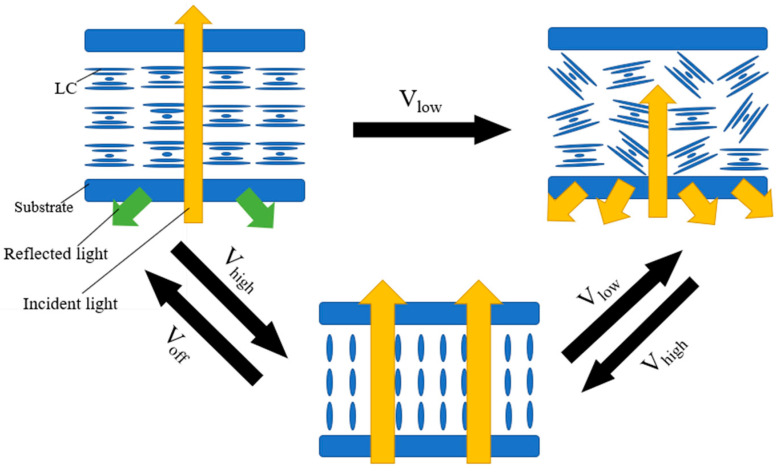
Schematic diagram of bistable mode switching for positive dielectric liquid crystals.

Liquid crystals with positive dielectric anisotropy constant, as a kind of low-cost and easy-to-acquire liquid crystals, have been used in a large number of bistable devices at an early stage. As shown in Figure 1, the switching of this type of device needs to rely on the homeotropic texture as a transition. When high voltage pulses are applied to the cholesteric liquid crystals, the dielectric effect leads to helical deconvolution and the liquid crystal molecules with homeotropic texture are aligned in parallel to the electric field. The devices can be stabilized in different optical states by controlling the length of time the electric field is turned off. If the electric field is turned off immediately, the liquid crystals will relax into the planar texture induced by the substrate parallel orientation treatment. On the contrary, when the electric field is turned off slowly, the liquid crystals relax into the focal conic texture. However, the Helfrich deformation can be used for fast response time from planar texture to focal conic texture. The switching can be performed without homeotropic texture under short voltage pulse (~10 ms) [25]. Rather than turning planar texture into focal conic texture through a nucleation process, the fast voltage pulse only deforms the cholesteric planar layers to form wrinkled layers. Nemati et al. found the effects of alignment layer on the Helfrich deformation. The alignment layer with a longer alkyl chain demonstrates shorter turn-on and -off response times (less than 10 ms) [26]. In addition, in order to reduce the defects of the planar texture, it is generally necessary to introduce parallel alignment on the substrates, which may lead to a slight decrease in the stability of the focal conic texture. In order to solve this problem, people have tried to introduce polymer networks. This method, first reported by West et al., introduces a 20 wt% polymer network in a liquid crystal system with positive dielectric anisotropy. Due to the anchoring force of the polymer network, liquid crystal molecules with focal conic texture can obtain permanent stability [27]. However, higher polymer content can result in excessive drive voltage. Switching from focal conic texture to planar texture requires driving voltages up to 130 V. To solve this problem, Yang et al. reduced the content of the polymer network to 5 wt%. Due to the reduced polymer content, the device can switch from focal conic texture to planar texture with only 50 V. At the same time, the focal conic texture still has permanent stability under this system [28]. In general, there have been many efforts on the bistable display mode of positive dielectric liquid crystals, and the bistable display performance is relatively easy to achieve, requiring only a short voltage pulse to switch between the two optical states.

**Figure 2:**
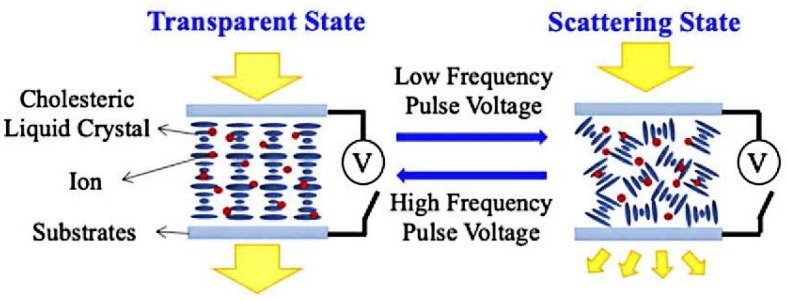
Schematic diagram of bistable mode switching for negative liquid crystals. Adapted from Ref. [29].

**Figure 3:**
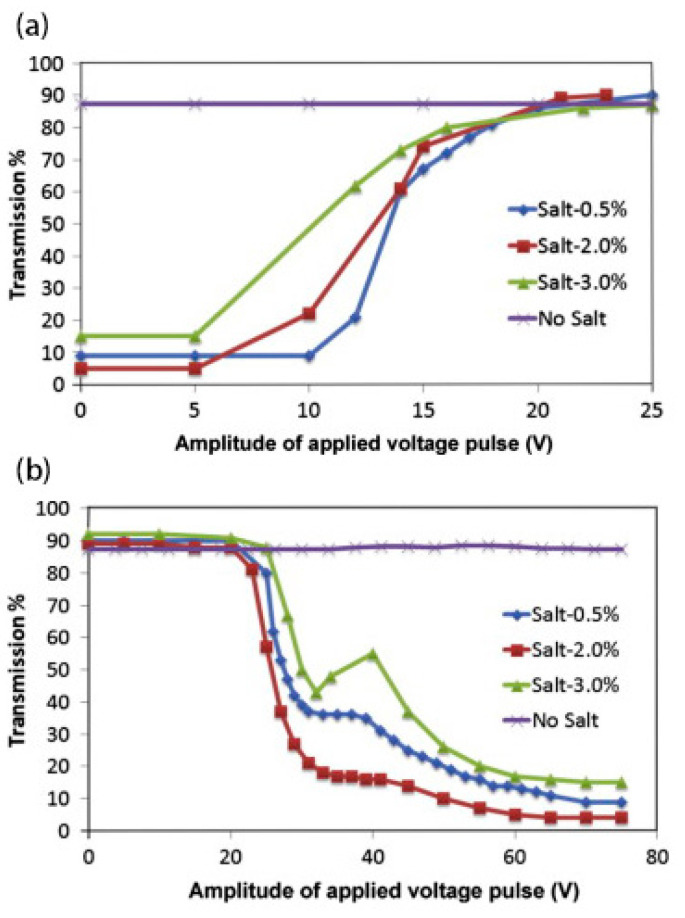
(**a**) Transmission at 0 V as a function of the amplitude of the applied voltage pulse with the frequency of 2 kHz and (**b**) transmission at 0 V as a function of the amplitude of the applied voltage pulse with the frequency of 60 Hz. Adapted from Ref. [31].

Negative dielectric liquid crystal devices are generally switched by the electrohydrodynamic effect and the dielectric effect. In order to obtain an obvious electrohydrodynamic effect, the devices often need to be doped with ions, as shown in Figure 2 [29]. When a low-frequency voltage is applied across the LC cell, it makes the ions to move along the electric field direction, namely, perpendicular to the cell substrate. The motion of the ions produces a turbulence and tends to align the liquid crystal along their moving direction [30]. This is the electrohydrodynamic effect. At this time, the liquid crystal molecules are in a dynamic scattering state. And the liquid crystals are stabilized in the focal conic texture when the electric field is turned off. At a high frequency, the ions cannot fellow the electric field due to their limited mobility. The aligning effect of the dielectric interaction is dominant. The overall effect of the applied voltage is to align the liquid crystal parallel to the device substrate [31]. And the liquid crystals are stabilized in the planar texture when the electric field is turned off. Moheghi et al. discovered the effect of salt on negative dielectric liquid crystal devices with bistable mode and prepared salt-doped cholesteric liquid crystals (SDCLCs) [29]. As shown in Figure 3, when a voltage is applied across the cell, a certain concentration of ions (about 2 wt%) causes a more obvious turbulence. At the same voltage frequency, it is easier for the device to obtain a stable scattering state [31]. Although the introduction of salt makes the scattering state easier to obtain, the applied voltage and ambient temperature can affect the scattering effect of the device. Lin et al. found the influence of voltage frequency and voltage amplitude on SDCLCs. And SDCLCs are more prone to dynamic scattering under the action of low-frequency and high-amplitude voltage. On the contrary, the aligning effect of the dielectric interaction is dominant The transmittance of the scattered state increases, resulting in a decrease in the contrast of the negative dielectric liquid crystal devices [29]. Sung et al. found that temperature is another factor having an influence on negative dielectric liquid crystal devices with bistable mode. At the same voltage frequency and amplitude, the light scattering becomes more pronounced as the temperature increases [32]. Therefore, the influence of external factors such as external electric field and temperature should be fully considered during the preparation of negative dielectric liquid crystal devices with bistable mode.

**Figure 4:**
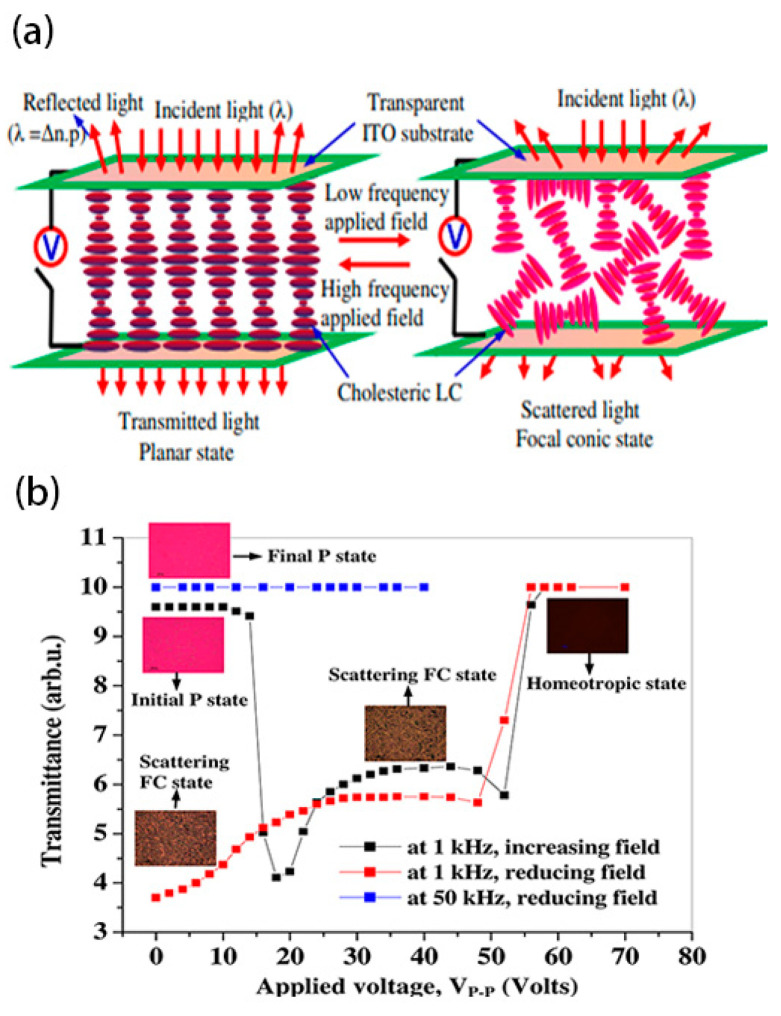
(**a**) Schematic diagram of bistable mode switching for dual frequency liquid crystals and (**b**) voltage dependent electro-optic transmittance of device at frequency 1 kHz and 50 kHz. The consequential microphotographs of the planar, focal conic, and homeotropic textures in transmissive mode under crossed polarizer at 200× are shown in the inset. Adapted from Ref. [33].

The switching method for bistable mode with dual-frequency liquid crystals is similar to that of negative dielectric liquid crystals, which is switched by high-frequency and low-frequency voltages, as shown in Figure 4 [33]. Due to the method mainly relying on the dielectric effect at different electric field frequencies, the device has both a faster response speed and a simpler switching performance [34].

**Figure 5:**
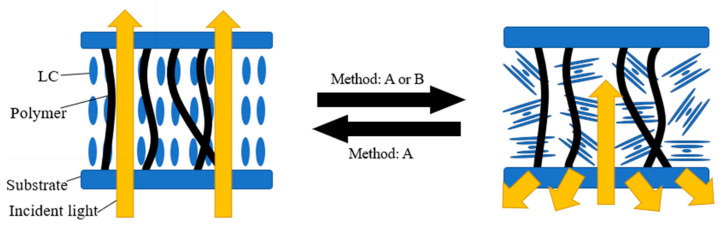
Schematic diagram of bistable mode switching with homeotropic texture (A and B are the dielectric effect and the electrohydrodynamic effect, respectively).

**Figure 6:**
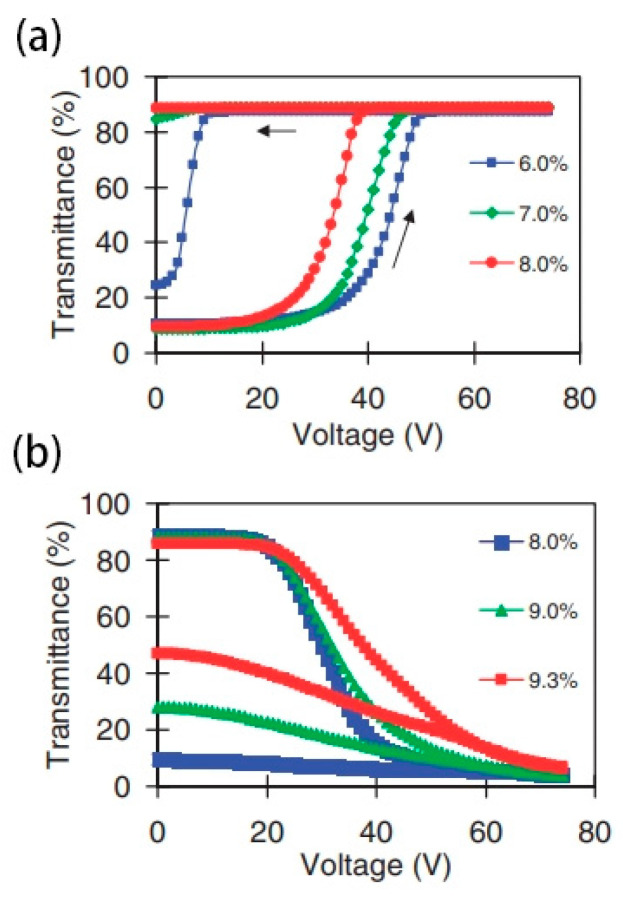
(**a**) The transmittance under applied voltage (100 Hz) curve of the devices with various polymer concentrations (where the upward arrow indicates the change in transmittance with increasing voltage. The arrow pointing to the left indicates that the device can stabilize at a certain transmittance after removing the electric field) and (**b**) the transmittance under applied voltage (20 kHz) curve of the devices with various polymer concentrations. Adapted from Ref. [35].

In addition to the above modes, it is also a common method to use homeotropic texture instead of planar texture as the transparent state of devices. The introduction of the polymer network is necessary to change the homeotropic texture from a transition state to a stabilized state, as shown in Figure 5. Ma et al. found the effect of polymer content on device switching. As shown in Figure 6, the high polymer content will cause the driving voltage of the device to be too high. On the contrary, too low polymer content will cause the liquid crystal molecules in the initial state difficult to maintain a good homeotropic orientation [35]. As is well known, the electrode area is a factor that affects the contrast of bistable LC devices. Li et al. reported a double-sided three-terminal electrode driving method, which can effectively improve the contrast of the device by appropriately reducing the electrode area [36]. Overall, the bistable mode LC device using the homeotropic texture has a relatively good application prospect. It should be noted that only by selecting the most suitable polymer content or in-plane electrode area can ideal electro-optical performance of bistable devices be achieved.

The advantage of bistable devices lies in their excellent stability. Liquid crystal molecules with planar texture or focal conic texture are in lower energy states. Without the stimulation of an external electric field, the directional direction is relatively stable. Bistable mode devices can be used in the field of smart windows and handwriting boards. However, bistable mode has only two stable textures, which will greatly limit its application in multimodal situations.

### 2.2. Tri-Stable Mode

**Figure 7:**
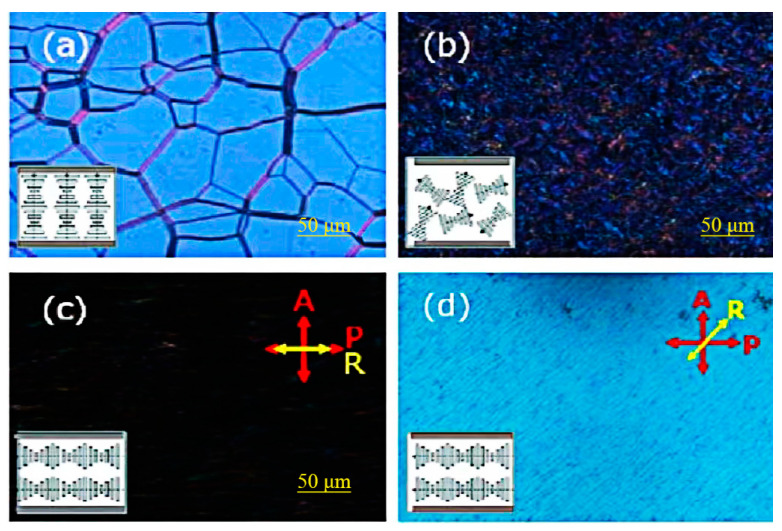
Textures of three stable states: (**a**) planar state, (**b**) focal conic state, (**c**,**d**) ULH state with optical axis at 0° and 45° with respect to the polarizer. Adapted from Ref. [39].

Cholesteric liquid crystal devices in tri-stable mode can usually switch electronically between three textures, that is, planar texture, focal conic texture, and fingerprint texture, as shown in Figure 7. When liquid crystal molecules are in planar and focal conic textures, the liquid crystal device in the tri-stable mode is in both transparent and opaque states [19,37]. The helical axes composed of liquid crystal molecules in the fingerprint texture are often preferentially parallel to the substrate in a certain direction due to substrate preorientation, showing a well-aligned uniform lying helix (ULH) texture. The axial direction of the helix in the ULH texture is parallel to the substrate, and the incident light can be transmitted through the device without selective reflection [38]. The transmittance of ULH texture is similar to that of homeotropic texture.

Compared with the bistable mode, the tri-stable mode has better display performance and can have multiple switching modes between different optical states in practical applications. However, the ULH texture is a metastable state that is difficult to obtain [40,41,42,43], so various switching methods have been continuously developed [44].

**Figure 8:**
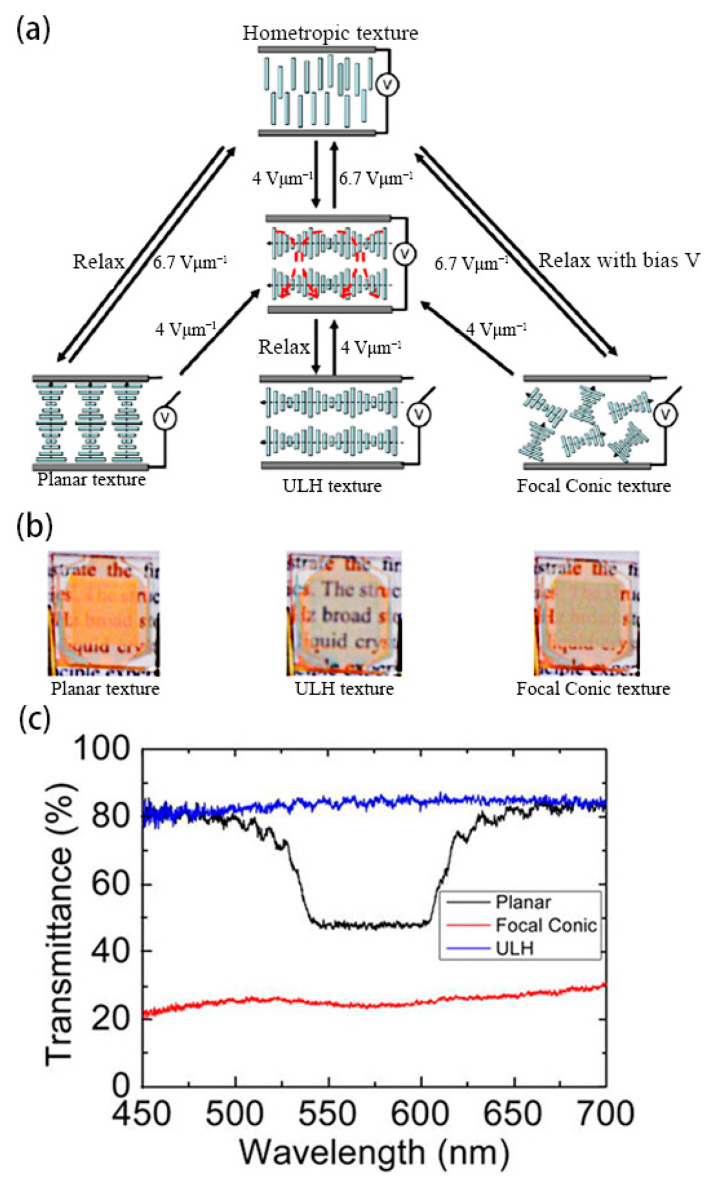
(**a**) Schematic diagram of device, (**b**) physical diagram of the tri-stable device, and (**c**) transmission-spectrum of three stable states. Adapted from Ref. [39].

Lee and Patel reported the earliest and most commonly used method for obtaining ULH texture. That is, when the liquid crystal is cooled from isotropy to the cholesteric phase, the cholesteric liquid crystal can be stabilized in ULH texture by using AC voltage [45,46]. Although the ULH texture obtained by this method is not defective, the cooling process will increase the switching time of the device, so it becomes important to explore the method of pure electric field switching [47]. After that, Inoue et al. found a mechanism for switching that relies on electrohydrodynamic effects, where the applied electric field induces a torque to orient the LC director perpendicularly to the substrates, while the shear induces a torque to orient the LC director along the shear flow. To achieve a good balance between their torques, the helix axis should be perpendicular to the shear flow. Meanwhile, the key to improving the helix-axis uniformity of the ULH texture lies in controlling the growth of the focal conic domains [38]. Wang et al. found that short pitch can effectively improve the stability of the ULH texture. Liquid crystals with short pitch have lower free energy in the ULH texture than liquid crystals with long pitch. As shown in Figure 8, liquid crystals with short pitch can switch to the ULH texture by electrohydrodynamic effects. Due to the short pitch of the cholesteric liquid crystals, the device has the ability to adjust color [39]. Nian et al. found that temperature, voltage amplitude, and voltage frequency are main factors affecting the texture stability of ULH. These three factors influence the stability of ULH through the effects of electrohydrodynamics. Usually, perfect ULH textures can be obtained at high-voltage amplitudes and low frequencies [43].

**Figure 9:**
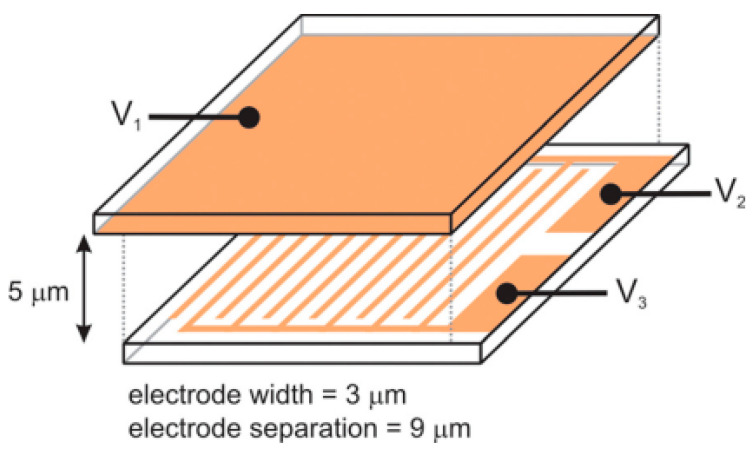
Schematic diagram of a liquid crystal cell with in-plane electrodes. Adapted from Ref. [50].

Electrohydrodynamic effect induction is an effective method for switching the ULH texture, and the effect often needs to occur at a certain ion concentration. However, ion migration and segregation induced by electric fields can cause the device to degrade over time, thereby reducing the service life of the device [48,49]. For this reason a pure dielectric effect switching method generated by in-plane electrodes has been proposed [2]. Gardiner et al. proposed an in-plane electric field switching method for three-electrode configurations, which can be realized by applying a low-frequency electric field at room temperature. This method avoids the competition between transparency and stability of devices caused by electrohydrodynamic effects [2]. As shown in Figure 9, the liquid crystals are in the planar texture in the initial state due to the reverse parallel pretreatment of the upper and lower substrates. When the in-plane electric field (V2 and V3) is applied, the liquid crystal texture appears defective. As the electric field increases, the defects become more uniform. After removing the electric field, the liquid crystal relaxes to the ULH texture [50]. Despite the simplicity of the method and the good effect of the ULH texture, the device preparation process is relatively more complicated. Yu et al. reported a dielectric effect switching method without an in-plane electric field. Choosing suitable liquid crystals to make the liquid crystals in the ULH texture uniaxial orientation between the upper and lower substrates is the key to the realization of this method. Under the action of the dielectric effect, the device can be stabilized in the ULH texture when the liquid crystals slowly decrease the voltage amplitude to zero at a certain rate from the homeotropic texture state at higher voltages [46,51].

In addition to the electrohydrodynamic and dielectric effects, the flexural electric effect can also effectively induce the ULH texture [52,53,54]. The flexoelectric effect tilts the helical axis and its intensity is linearly related to the electric field strength. For most traditional rod-shaped liquid crystals, the flexoelectric and dielectric effects are usually coupled under the influence of an electric field and strongly depend on the dielectric anisotropy. In order to suppress the dielectric effect and highlight the flexoelectric effect, doping bent-core liquid crystals is an effective method. This method can significantly reduce the dielectric anisotropy of the system and obtain a good switching effect [39]. Analogous to rod-shaped molecules, bent-core molecules also exhibit nematic phases. Typically, bent-shaped liquid crystalline molecules are composed of three units: an angular central core, two linear rigid arms, and terminal chains [55,56,57,58,59,60,61]. Bent-core dimers with a flexible spacer consisting of an odd number of methylene groups are a kind of typical bent-core liquid crystal which are usually synthesized by using rod-shaped liquid crystals [62]. Lin et al. explored the flexoelectric-effect-induced switching mechanism of positive dielectric cholesteric liquid crystals doped with the bent-core liquid crystal dimer CB7CB. Due to the introduction of CB7CB, the device can easily switch from focal conic texture or planar texture to ULH texture [63].

In general, obtaining stable and good ULH texture is the key to preparing tri-stable devices. Due to the introduction of the ULH texture, this device adds a responsive optical stable state, which makes it more practical and adjustable, and has bright prospects in fields such as smart windows and liquid crystal gratings. Nevertheless, multi-stable devices are both challenges and opportunities for researchers, and the response performance of new tri-stable or multi-stable modes is still further developing.

### 2.3. Multi-Stable Mode

The multi-stable mode generally adds a stable state on the basis of the tri-stable mode. The devices in this mode have stronger response performance. Lu et al. reported a multi-stable mode device for the first time. In this mode, the device has four stable states: planar texture, focal conic texture, ULH texture, and the broadband reflection state. For the switching method of the broadband reflection state, the author draws on the report of Hu et al. The introduction of anionic chiral ionic liquids containing chiral groups to the ChLC system is the key to the preparation method. Under the action of an applied DC electric field, the anion in the ionic liquid moves toward the positive pole and causes a higher concentration of the chiral group near the positive pole and a lower concentration near the negative pole, resulting in a concentration gradient of the chiral compound. When the high-frequency AC voltage is applied, the reflected wavelength can cover the entire visible wavelength band. At the same time, when a reverse applied DC electric field is applied, the state of the broadband reflection disappears. Except for the broadband reflection state, the switching mode of the other three states is the same as the switching mode of the tri-stable state in the previous section [18,64].

Because of the ability of broadband reflection, devices with multi-stable mode can be used in the field of smart windows with thermal radiation shielding. However, the function of the multi-stable mode is stronger than that of the tri-stable mode. However, there are few studies on the multi-stable mode. The future research of this mode can pay more attention to the improvement of device stability and the broadband reflection effect.

## 3. Performance Optimization

In recent years, the multi-stable cholesteric liquid crystal optical devices are becoming more and more mature, but the disadvantages of low contrast, high switching time, and low mechanical strength hinder their commercialization.

### 3.1. Optimization of Contrast Ratio

**Figure 10:**
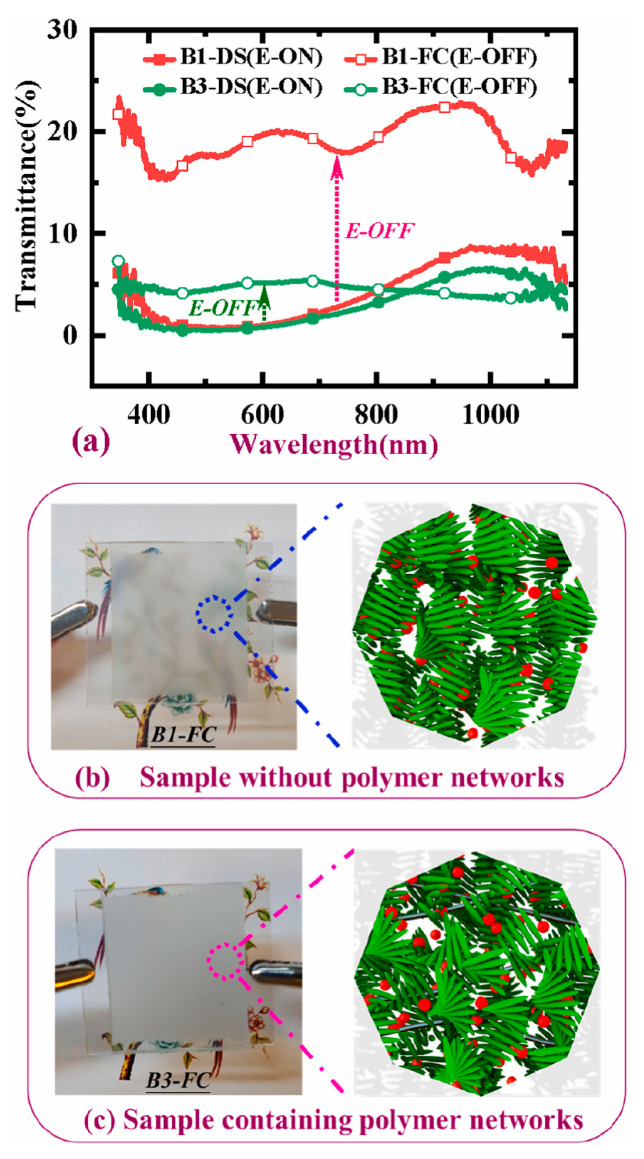
(**a**) Transmission spectra of the sample B1 without polymer networks and the sample B3 with polymer networks, (**b**) photographs of the opaque state and schematic diagrams of the focal conic state for sample B1 without polymer networks, (**c**) photographs of the opaque state and schematic diagrams of the focal conic state for sample B3 with polymer networks. Adapted from Ref. [66].

In the field of liquid crystal optical devices, contrast ratio generally refers to the ratio of maximum transmittance to minimum transmittance. The main reason for the low contrast of the device is the poor scattering ability of the liquid crystal in the focal conic texture. To solve this problem, the introduction of a polymer network is a viable approach. Liang et al. reported an electro-thermal switchable bistable reverse-mode polymer-stabilized cholesteric texture light shutter. The introduction of a polymer network can enable liquid crystal molecules to produce more disordered microdomains while simultaneously stabilizing them, resulting in a stronger light-scattering state. The principle lies in the deformation of the polymer network due to a high-voltage pulse, which produces a strong anchoring force on the focal conic texture. However, only by using the thermal annealing method to decrease the aligning effect of the polymer network can the liquid crystal be reverted to the planar texture. This is obviously not conducive to the wide application of the device [16]. In order to obtain pure electrical response of multi-stable devices with polymer, He et al. reported a method to drive polymer-stabilized bistable devices using electrohydrodynamic effects. Compared with Liang et al.‘s report, this method only needs to apply high-frequency and low-frequency electric fields to complete all operations. At the same time, the concentration of polymer network also affects the contrast of the device. With the increase of polymer content, the transmittance of the focal conic texture decreases and the contrast of the device increases. However, the polymer content must be controlled within the critical range, beyond which the ordered planar arrangement of the liquid crystal is disturbed by a dense polymer network, and the disorder increases as the polymer concentration increases. This will result in a decrease in the maximum transmittance of the device and a decrease in the contrast of the device [65]. Li et al. reported a bistable ion-doped cholesteric liquid crystal smart window with a small amount of polymer. Compared with He et al.‘s report, due to the small polymer content (about 0.5 wt%), the critical operating voltages of the device in this report are significantly reduced. This is conducive to improving the energy saving and safety of the device. As shown in Figure 10, the minimum transmittance of the device remains at a desirable level due to the combined effect of the ions and the polymer network, in which ions enhance the electrohydrodynamic effect [66].

**Figure 11:**
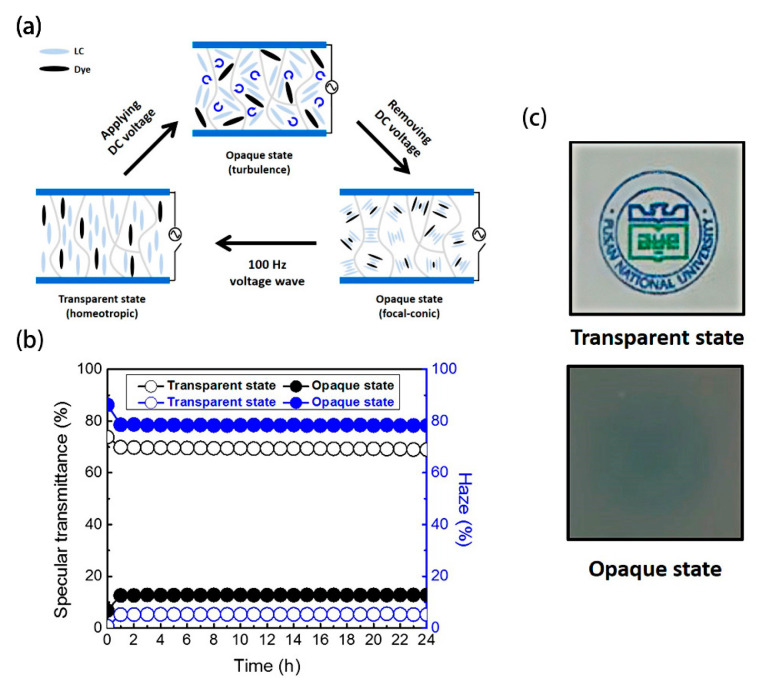
(**a**) Schematic diagram of devices, (**b**) the change of haze value and specular transmittance with time of the device, and (**c**) photographs of the fabricated ion-doped CLC cell placed on printed paper. Adapted from Ref. [74].

In addition to the introduction of polymer networks, doping dichroic dyes is also a common way to improve contrast. The main function of dichroic dyes is to reduce the minimum transmittance of the devices by absorbing incident light. When the absorption axes of the dye molecules and the polarization of the incident light are aligned, dye molecules strongly absorb the incident light. On the other hand, dye molecules weakly absorb the incident light when the axes of the dye molecules and the polarization of the incident light are crossed with each other. Dichroic dyes are convenient for use in the control of light transmission through an LC cell because dye molecules are easily oriented along the LC molecules [67,68,69,70]. Yu et al. report an S-428 dichroic dye-doped bistable device in which the liquid crystal can switch between the planar texture and the focal conic texture. Due to the combined effect of absorption by dichroic dyes and scattering by liquid crystals, the transmittance of a device with focal conic texture is greatly reduced [25]. Gahrotra et al. showed that in addition to improving contrast, doped dichroic dyes can reduce the critical operating voltages (*V_c_*). Here, according to de Gennes’s theory in the bistable switching of CLCs, *V_c_* can be expressed as

(1) $$V_{c}=(\pi^{2}\cdot d\cdot HTP\cdot C)\sqrt{\frac{k_{22}}{∆\varepsilon }}$$

where $k_{22}$, d, C and ∆ε are the twist elastic constant, cell gap, chiral concentration and the dielectric anisotropy of the LC, respectively. And the HTP stands for the helical twisting power of the chiral agent. Due to the dye molecules exhibiting a liquid crystalline nature, the total LC content will be increased and the chiral concentration will be reduced with the introduction of dichroic dyes. At the same time, since the chiral concentration is proportional to the critical operating voltages, the latter will also show a decreasing trend [71,72]. Although the introduction of dichroic dyes can increase the contrast of the device, dichroic dyes can reduce the maximum transmittance to some extent in the system with planar texture. This is because the dichroic dye molecules are parallel oriented along the liquid crystal molecules, and the incident light is obviously absorbed by the dye molecules. In order to solve this problem, it is an effective method to introduce polymer network and dichroic dye molecules simultaneously. Huh et al. compared the electro-optical performance of different patterned electrode devices doped with dyes, and the cross-patterned electrode arrangement has superior electro-optical performance compared to the parallel-patterned electrode arrangement and the unaligned parallel-patterned electrode arrangement [73]. Kim et al. reported a polymer-stabilized dye-doped device, which was driven by electrohydrodynamic effects unlike the in-plane electrode driving method reported by Huh et al., as shown in Figure 11. The electrohydrodynamic-effect-driven device has a more uniform focal conic texture of the liquid crystals compared to the in-plane electrode-driven device. This results in higher device haze and contrast [74].

In addition to the introduction of polymer networks and dichroic dyes, the gap size of the cell and size of the pitch also have influence on the contrast. As we all know, d/p is one of the important parameters that affect the electro-optical performance of liquid crystal devices (where d and p are the cell gap and pitch). When the d/p is too large, the device is easy to stabilize in the focal conic texture. On the contrary, the device is easy to stabilize in the planar texture. Being too stable in either texture will cause the optical properties of the other texture to decline. Therefore, when d/p is in a moderate range, the devices have the maximum contrast [75,76,77].

The introduction of polymer networks and dichroic dyes can help to improve the contrast of the devices. When introducing polymer networks, care should be taken that the polymer content should not be too large. Too much polymer will cause the liquid crystal molecules to be subjected to too much anchoring force, which will cause the multi-stable devices to lose stable mode. The introduction of dichroic dyes can not only improve the contrast but also improve the driving voltage of the device. Moreover, the introduction of dichroic dye and polymer networks can improve the contrast of the device more effectively. In addition, the d/p value is also an important factor affecting contrast. In general, multi-stable devices have the greatest contrast when d/p is in the moderate range.

### 3.2. Optimization of Switching Time

Switching time generally refers to the time when the transmittance of the device increases from 10% to 90% of the maximum value and vice versa. Compared to the switching time of traditional liquid crystal devices (about 10 ms), the switching of multi-stable devices with formal liquid crystals requires 300 ms. This problem is not conducive to the application of the device in the field of display. In general, it is believed that the problem is mainly caused by the homeotropic texture experienced by the liquid crystal when it switches from a focal conic texture to a planar texture.

**Figure 12:**
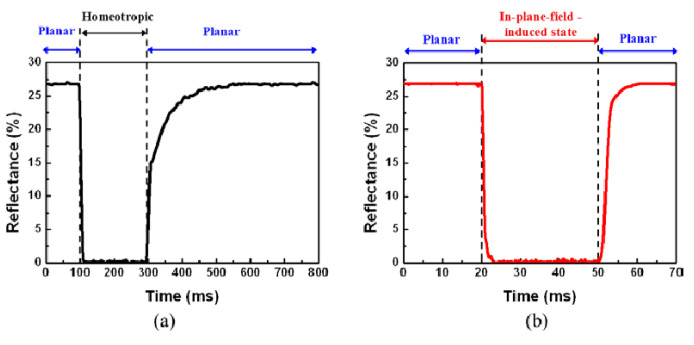
(**a**) Vertical switching between the planar and homeotropic states and (**b**) in-plane switching between the planar and in-plane-field-induced states. Adapted from Ref. [79].

Kim and Oh et al. reported three- and four-terminal in-plane electrode structures, respectively. Under the action of in-plane electrodes, the liquid crystal molecules with focal conic texture are uncoiled in the direction of the electric field and form an in-plane-field-induced state. When the electric field is turned off, the unwound liquid crystal molecules can form a helical structure through a simple rotation in the same plane. As shown in Figure 12, this method with in-plane electrode greatly reduces the switching time, and it only takes 50 ms to complete the switching operation [78,79].

In addition to the above methods, the introduction of dual-frequency liquid crystal can also effectively reduce the switching time. Hsiao shows that when the pitch is at the right size, the device with dual-frequency liquid crystal can switch from the focal conic texture to the planar texture in only 10 ms. This process is much faster than the transition time of liquid crystals with positive dielectric anisotropy (about 300 ms). Liu et al. synthesized a ferroelectric liquid crystal, which was used as a chiral dopant and combined with a dual-frequency liquid crystal to prepare a bistable device. The device could significantly reduce the response time to 1.5 ms while lowering the driving voltage [80]. Although this switching method has significant advantages, this method is limited by the high cost of dual-frequency liquid crystal.

Yu et al. explored the factors that affect the switching time. When the liquid crystal molecules switch from the homeotropic texture to the planar texture, they will experience a transition planar texture, and the pitch of this texture is longer than the initial planar texture. With the extension of time, the pitch of the transition planar texture returns to the initial planar texture pitch. In this process, the elastic constants are key influencing factors [81], as shown in the following equation:

(2) $$P’=P({sin}^{2}\theta +\frac{K_{33}}{K_{22}}{cos}^{2}\theta )$$

Here, the polar angle *θ* is the angle between the direction of the LC molecules and the helical axis, while *K*_33_ is the bend elastic constant and *K*_22_ is the twist elastic constant. For most commercially available nematic liquid crystals, they have *K*_33_*/K*_22_ > 1. This will result in the transition pitch (*P’*) always being greater than the initial pitch (*P*). For this reason, the introduction of dimer liquid crystals to reduce *K*_33_ is also an effective method. When *K*_33_*/K*_22_ = 1, *P’* is the same as *P*, and the switching time of the device will be reduced to about 16 ms. Lu et al. introduced dimers into bistable systems doped with dichroic dyes. And the introduction of dimers can significantly offset the increase in switching time caused by dye doping [82].

The introduction of in-plane electrodes, dual-frequency liquid crystals, and dimers can solve the problem of the long switching time of multi-stable devices with positive dielectric liquid crystals. All the three methods reduce the switching time by avoiding the occurrence of homeotropic texture or inhibiting the occurrence of transitional planar texture. Among them, the introduction of the dual-frequency liquid crystals method has the most obvious effect, and the switching time can be controlled at about 1.6 ms.

### 3.3. Optimization of Mechanical Properties

**Figure 13:**
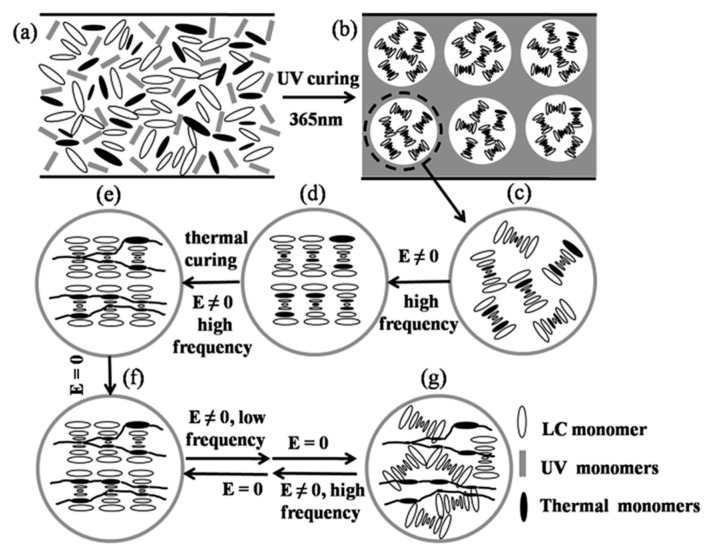
Schematic diagram of the two-step polymerization process. (**a**) The initial state of the liquid crystals mixture, (**b**) the non-liquid crystal monomers are polymerized under ultraviolet light, (**c**) the liquid crystal molecules in the polymer are in focal conic texture, (**d**) the liquid crystal molecules are in planar texture under high frequency electric field, (**e**) the liquid crystal monomers are polymerized with the increase of temperature, (**f**) after the electric field is removed, the liquid crystals with planar texture remain stable under polymer anchoring force, (**g**) at low frequency electric field, liquid crystal molecules switch from planar texture to focal conic texture and this state can remain stable after the electric field is removed. Adapted from Ref. [83].

**Figure 14:**
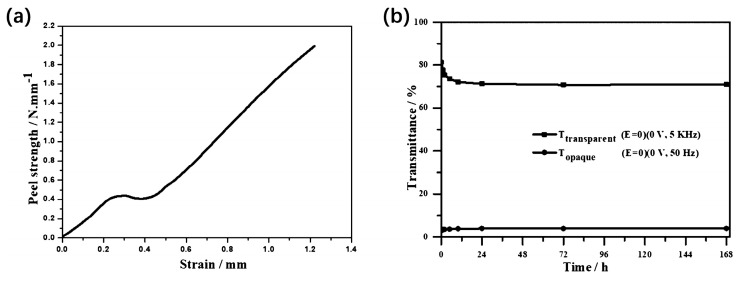
(**a**) Mechanical properties and (**b**) stability test. Adapted from Ref. [84].

In addition to the problem of contrast and switching time, the poor mechanical properties of liquid crystal materials generally lead to poor impact resistance of the device, where it is not easy to prepare a large area, and unstable optical properties of the device after long-term use. A common strategy in current research is to increase the polymer content in the device. Wang et al. reported a two-step polymerization method combining UV polymerization and thermal polymerization, as shown in Figure 13, in which the polymer formed by the thermal polymerization of liquid-crystalline monomers ensures the memory effect of the orientation order of the liquid crystal molecules, and the polymer formed by the UV polymerization of isotropic monomers enhances the mechanical properties of the device [83]. Hu et al. report a method for stabilization of a polymer framework, which is prepared by thermal curing of an epoxy resin with two thiols. The liquid crystal molecules can be maintained in the planar/focal conic texture for a long time after the removal of the electric field with the anchoring of the polymer framework. It is worth mentioning that the polymer framework consists of close to forty percent polymer content, resulting in devices with better mechanical properties than those reported by Wang et al. As shown in Figure 14, the peel strength of the device with a polymer framework reached 2.0 kN/m. At the same time, the device has a contrast ratio of more than 20 and can remain stable for 168 h [84]. Miao et al. reported a photomask stepwise UV photopolymerization method and prepared bistable devices with polymer spacer columns. The mechanical properties of the devices were significantly improved due to the support of the spacer columns. And the introduction of the polymer spacer columns also resulted in devices with more excellent contrast and driving voltage [85]. In general, the introduction of non-liquid crystalline polymer networks is the key to improving the mechanical properties of devices. At the same time, in the preparation process, it is usually necessary to adopt the method of multi-step curing or masking to ensure the stability of the multi-stable mode.

## 4. Application

Multi-stable cholesteric liquid crystal devices have been continuously developed and optimized. They exhibit increasingly excellent electronic control optical performance and can be applied in multiple fields such as smart windows, handwriting boards, liquid crystal fibers, and light valves [24,86,87,88,89].

**Figure 15:**
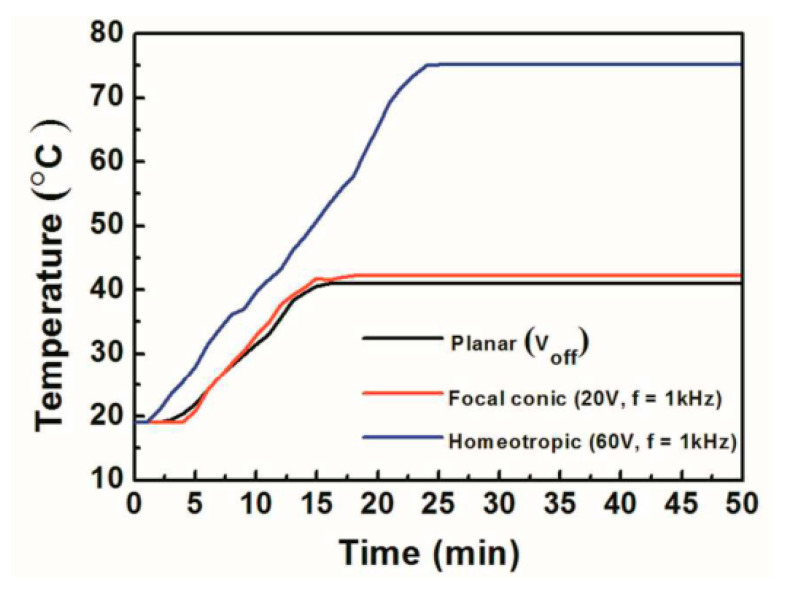
Temperature variation with time in dye-doped bistable devices under different textures. Adapted from Ref. [86].

**Figure 16:**
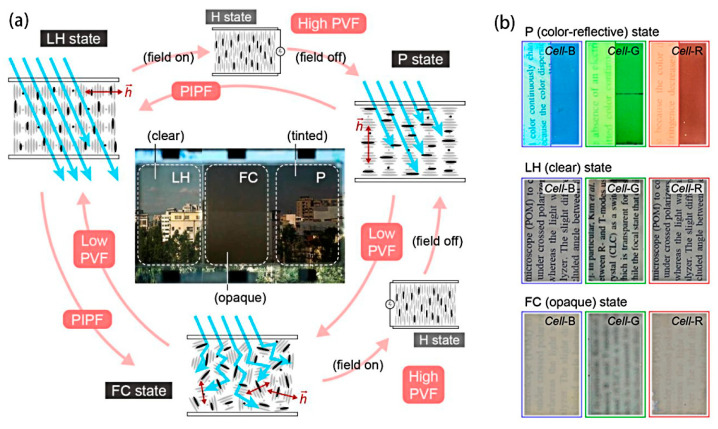
(**a**) Schematic diagram of dye-doped tri-stable devices and (**b**) physical diagram of colored mode devices. Adapted from Ref. [2].

It is well known that near-infrared (NIR) light accounts for about 50% of the total energy of direct sunlight. In the hot summer, the inability of ordinary windows to block the incoming near-infrared (NIR) light leads to high indoor temperatures and increases the energy consumption of air-conditioning and other equipment. However, planar-texture cholesteric liquid crystals with appropriate pitch can selectively reflect some of the NIR light with similar transparency as ordinary glass, which will greatly reduce energy consumption and improve the comfort of the occupants. Lee et al. explored the thermal insulation effect of dye-doped cholesteric liquid crystal bistable devices. The authors fabricated a “miniature house” with liquid crystal smart windows and irradiated it with a 1064 nm laser beam, in which the reflected wavelength of the cholesteric liquid crystals was in the near-infrared region. As shown in Figure 15, the internal temperature of the “house” is similar to that of the focal conic texture and much lower than that of the homeotropic texture when the liquid crystals are in the planar texture. This suggests that the reflective ability of liquid crystals in the planar texture for infrared light can well reduce the temperature inside the house [86]. Du et al. demonstrated a bistable device with a bilayer structure of cholesteric liquid crystals and chiral polymer membranes, in which the chiral polymer membranes reflect optical rotation in the opposite direction of the cholesteric-phase optical rotation but in the same wavelength range. The device has super-reflective ability to near-infrared light and has great application potential in the field of temperature control [24]. The tri-stable devices also can be used in the field of smart windows. Like bistable devices, tri-stable devices also have the ability to reflect near-infrared light. However, the difference is that the tri-stable devices have one more ULH texture than the bistable devices, in which the helical axis of the cholesteric liquid crystals is parallel to the substrate, and all wavelength ranges of incident light can pass through the devices, which makes the devices have stronger response performance. In addition to the function of shielding infrared, the multi-stable smart window can also switch colors. Li et al. reported a device with pulsed in-plane fields (PIPF) combined with pulsed vertical fields (PVF), which has the ability to regulate color. As shown in Figure 16a, since the doped dichroic dye has the same orientation as the liquid crystal, this allows the device to have three modes of transparent, black and transparent, and black and opaque. Notably, planar texture can be used as a glare reduction mode due to the dye’s enhanced absorption of incident light. At the same time, the author introduced the color-changing ability into the device by controlling the cholesteric liquid crystals pitch within the wavelength range of visible light and demonstrated a color-tunable glass, as shown in Figure 16b [2].

In addition to smart windows, devices are often used in the fields of handwriting boards and liquid crystal fibers [87,88,89]. The reflection wavelength of cholesteric liquid crystal in handwriting boards is in the visible light region. The device shows “handwriting” in planar texture and “no handwriting” in focal conic texture, in which the electrical drive plays the role of “wiping” the handwriting. Xing et al. developed a bistable liquid crystal handwriting board based on polymer-dispersed cholesteric liquid crystals. Xing et al. developed a bistable liquid crystal handwriting board based on polymer-dispersed cholesteric liquid crystals, and explored the effects of liquid crystal droplet size and polymer network morphology on the electro-optical properties of the handwriting board [88]. Miao et al. reported a liquid crystal handwriting board that can locally erase handwriting using infrared light by doping modified carbon nanotubes and explored the effect of doping content on electro-optical properties [87]. Wang et al. reported a color-tunable liquid crystal fiber, which was constructed by mechanical stretching and UV polymerization with good thermal and mechanical stability. Under the stimulation of an electric field, the liquid crystal fiber has three states, and the fiber has considerable potential for applications in the fields of color-tunable clothing, flexible smart fabrics, and military camouflage [89]. In addition to the above two fields, tri-stable devices can also be applied in the field of gratings. Xing et al. studied the dynamics of the switching process of liquid crystal grating by digitally processing recorded image sequences and reported a tunable liquid crystal grating [90].

## 5. Conclusions

In conclusion, the multi-stable devices only apply an electric field during switching and do not require a continuous electric field to maintain the various optical states of the device, so the multi-stable device has lower energy consumption than traditional liquid crystals optical devices. At present, the modes of electrically driven multi-stable cholesteric liquid crystal devices have been studied: bistable mode, tri-stable mode, and multi-stable mode. Bistable mode is the most basic mode, which has only two states of transparency and scattering. The tri-stable mode introduces the ULH texture on the basis of the bistable mode, and the device has three states of scattering, fully transparent and transparent with selective reflection. Multi-stable mode can be prepared by doping a chiral ionic liquid in a tri-stable mode, which has scattering, fully transparent, transparent with selective reflection, and broadband reflection states. Despite the increasing response performance of devices, each drive mode has different issues that affect the electro-optical performance of the devices. The field of bistable devices mainly focuses on the modification of contrast, switching time and mechanical properties. Modification methods can be adopted by doping dichroic dyes and polymers. The problem of tri-stable devices is that the ULH texture is not easy to obtain and difficult to stabilize for a long time. This problem can be solved by applying different switching methods. At present, there are few studies on multi-stable mode devices, and the switching method is relatively simple. Therefore, for the four-stable state, the subsequent research should pay more attention to the application of the device, try to expand the broadband reflection range, and try to achieve the ability of hyper-reflection in this mode. Overall, the electro-optical performance of liquid crystal devices, such as driving voltage, contrast, and response time, still needs to be continuously improved before practical applications, and good mechanical properties, wearability, and foldability are required for practical applications, which are also one of the future development directions of multi-stable devices. Due to the advantages of low energy consumption for switching between optical states and no need for energy consumption to maintain each optical state, multi-stable devices have broad application prospects, such as infrared shielding, electrohydrodynamic effects, and responsive displays. This is both a challenge and an opportunity for researchers, and the response performance of new bistable, tri-stable, or multi-stable modes still needs to be further developed.

## Data Availability

Not applicable.
